# Spin exchange broadening of magnetic resonance lines in a high-sensitivity rotating K-Rb-^21^Ne co-magnetometer

**DOI:** 10.1038/srep36547

**Published:** 2016-11-10

**Authors:** Yao Chen, Wei Quan, Sheng Zou, Yan Lu, Lihong Duan, Yang Li, Hong Zhang, Ming Ding, Jiancheng Fang

**Affiliations:** 1 School of Instrument Science and Opto-Electronics Engineering, Beihang University, Beijing 100191, China; 2 School of Instrument Science and Engineering, Southeast University, Nanjing 210096, China

## Abstract

Atomic co-magnetometers can be utilized for high-precision angular velocity sensing or fundamental physics tests. The sensitivity of a co-magnetometer determines the angle random walk of an angular velocity sensor and the detection limit for a fundamental physics test. A high-sensitivity K-Rb-^21^Ne co-magnetometer, which is utilized for angular velocity sensing, is presented in this paper. A new type of spin relaxation of Rb atom spins, which can broaden the zero-field magnetic resonance lines of the co-magnetometer, is discovered. Further studies show that the spin relaxation of Rb atoms is caused by a high Rb electron magnetization field. With this discovery, the total relaxation rate of Rb atoms is optimized to improve the sensitivity of the co-magnetometer. Moreover, its sensitivity is optimized by suppressing various noises. Especially, to suppress laser-related noises, the co-magnetometer is designed such that the sensitive axis of the co-magnetometer can be fixed to the direction in which the projection input of the earth’s rotation is 0. This is called a rotating co-magnetometer. A magnetic field sensitivity of 1.0 fT/Hz^−1/2^@5 Hz, which is equal to an angular velocity sensitivity of 2.1 × 10^−8^ rad s^−1^ Hz^−1/2^@5 Hz, is demonstrated using a spherical vapour cell with a diameter of 14 mm.

Atomic co-magnetometers use two spin ensembles occupying the same volume to suppress their sensitivity to magnetic field noise and maintain the sensitivity to rotations^1^,^2^, anomalous spin coupling with space anisotropy^3^,^4^, anomalous spin forces^5^,^6^,^7^, etc. In a K-Rb-^21^Ne co-magnetometer, the alkali spin ensemble couples with the noble gas spin ensemble through spin exchange interaction^8^, and alkali atoms are in the spin exchange relaxation free (SERF) regime^9^ to sensitively detect the direction of the ^21^Ne nuclear spin.

The SERF regime has been extensively studied for many years^10^,^11^. Spin exchange relaxation was always neglected in atomic sensors based on SERF. For example, spin exchange relaxation vanishes in a K-^3^He co-magnetometer and in SERF magnetometers^12^,^13^. In a K-Rb-^21^Ne co-magnetometer, Rb atoms are supposed to be in the SERF regime, and thus the spin exchange relaxation rate of Rb atoms is zero. However, we discovered a large spin exchange relaxation rate of Rb atoms, which is caused by a large Rb magnetization field that is in turn due to spin exchange interaction between Rb atoms and ^21^Ne atoms^14^. The Rb magnetization field is about 100 times larger than that of a K-^3^He co-magnetometer, and it can sufficiently broaden the zero-field magnetic resonance lines. We modelled the relaxation rates of Rb atoms and measured those of Rb atoms using the zero-field resonance lines of the co-magnetometer. The theoretical simulation and the experimental results show that the spin exchange relaxation rate is sufficiently increased by the Rb magnetization field. Moreover, we conclude that the spin exchange relaxation rate of Rb atoms can be reduced by half by changing nature enriched ^87^Rb and ^85^Rb atoms into pure ^87^Rb atoms in the current co-magnetometer arrangement. After the study on the relaxation rate, we optimized the total relaxation rate of Rb atoms by changing the pumping power density to maximize the response of the co-magnetometer and thus to improve its sensitivity.

An atomic spin co-magnetometer based on K-^3^He was developed for angular velocity sensing, and a sensitivity of 5 × 10^−7^ rad s^−1^ Hz^−1/2^ was achieved by Kornack and Romalis *et al*.^1^. Owing to a small gyro-magnetic ratio of a ^21^Ne atom, which is about 10 times smaller than that of a ^3^He atom, theoretical simulation showed that the fundamental rotation rate sensitivity of a Rb-^21^Ne co-magnetometer was about 10 times higher than that of a K-^3^He co-magnetometer at the same magnetic field noise level^1^. A magnetic field sensitivity of 1.0 fT Hz^−1/2^@5 Hz, which is equal to an angular velocity sensitivity of 2.1 × 10^−8^ rad s^−1^ Hz^−1/2^@5 Hz, was achieved in this study. To further improve the sensitivity, various noises were suppressed other than the response was maximized. The co-magnetometer uses Rb and ^21^Ne spins coupled together to suppress its sensitivity to magnetic field noise. The suppression factor characterizes the ability of magnetic field noise suppression. We optimized the suppression factors by optimizing the temperature of the vapour cell. Other noises, such as pumping power and probe laser power density fluctuations, influence the sensitivity. We suppressed these noises by reducing systematic errors in the co-magnetometer. Details of the optimization and noise suppression are shown in the results’ section. A high sensitivity of the co-magnetometer also ensures its application to anomalous spin force detection^6^,^7^.

The co-magnetometer is designed in configuration to improve the sensitivity. Traditionally, the apparatus is set up on an air-floated optical table^2^,^15^, and the sensing axis is vertical to the horizontal plane. In this paper, in order to suppress the input rotation rate of the earth to improve the sensitivity and calibrate the co-magnetometer by the earth’s rotation, the sensitive axis is in the y-direction in Fig. 1 and the whole apparatus can rotate around the z-axis; thus, the direction of the sensitive axis can change. All electronics and optics are configured on a directive drive rotary platform. In Fig. 2, owing to the projection of the earth’s rotation rate in the y-direction, the rotation rate in the y-direction changes as the whole apparatus rotates around the z-axis. The rotation rate input can be reduced to approximately 0 by fixing the sensitive axis around the east direction, as the co-magnetometer is mostly sensitive with an input of 0. The North Star is used to find the east direction. The details are illustrated in Fig. 2.

The basic principle of the co-magnetometer is similar to that of a K-^3^He co-magnetometer^1^,^16^. The schematic of the experimental setup is shown in Fig. 1. The co-magnetometer consists of a spherical aluminosilicate glass vapour cell with a diameter of 14 mm that contains a small droplet of a K-Rb (^85^Rb (72.2%) and ^87^Rb (27.8%)) mixture, ^21^Ne gas (70% isotope enriched) under a pressure of about 3 Atm, and N_2_ gas under about 40 Torr for quenching. The mole fraction ratio of K in the mixture is approximately 0.05. The cell wall has a thickness of approximately 0.2 mm. The cell was heated using a homemade 110 kHz AC electrical heater, which was pasted on an oven composed of boron nitride ceramic. The oven was in vacuum for thermal insulation. A 0.1 Pa vacuum was achieved using 10 L/s turbo molecular pumps. Four layers of *μ*-metal magnetic field shields and a layer of 10-mm-thick ferrite^17^ were used to shield the earth’s magnetic field. The diameter of the innermost ferrite layer was 100 mm. The residual magnetic field in the shields was further compensated by implementing a three-axis Helmholtz coil. The residual magnetic field in the shields was less than 2 nT after degaussing before compensation using the coil. A cooling water jacket was utilized to reduce the temperature of the vacuum wall. The temperature of the magnetic field shield was sufficiently reduced by means of the cooling jacket. The K-Rb hybrid pumping technique was utilized in the K-Rb-^21^Ne co-magnetometer^3^. K atoms were directly polarized by pumping light whose wavelength was locked on the D1 line of K atoms by a dichroic atomic vapour laser lock (DAVLL). The output light power from a tapered amplifier (TA) was about 1 W. The gauss shape pump beam was expanded, and the relatively uniform light intensity area of the beam was selected using a rectangular aperture to be approximately 12 mm × 15 mm to nearly fully cover the vapour cell. After passing the vapour cell, the pumping light was reflected by a mirror again to increase the power in the vapour cell because the hybrid pumping technique needs a high pumping power. Through spin exchange, Rb atoms were pumped by K atoms and ^21^Ne atoms were hyperpolarized by Rb atoms. If there was a rotation rate input, ^21^Ne atom spins would rotate at an angle from the z-axis. ^21^Ne atoms would produce a magnetic field perpendicular to the z-axis^8^, and it would be experienced by Rb atoms. Rb spins were used to sensitively detect the magnetic field, and those in the x-direction would change if the input ^21^Ne magnetic field was not 0. A linear polarized probe beam would rotate at an angle after passing Rb spins polarized in the x-direction. We measured the rotation of the probe beam to obtain the rotation rates. The probe distributed feedback (DFB) laser was detuned approximately 0.4 nm away from the absorption centre of the Rb D1 line. The photo-elastic modulation (PEM) technique was utilized to measure the rotation of the polarization plane of the linear polarized light. A noise eater composed of a liquid crystal retardance and a polarizer was used to stabilize the power of the probe light. The air density fluctuation caused by the temperature difference of the oven and room was reduced by enclosing all the laser beams in a bell jar pumped using a rotary pump. A rotary vacuum seal was utilized to keep the vacuum pumping as the whole apparatus rotated by the directive drive rotary platform.

## Results

### Spin exchange broadening of Rb zero magnetic field resonance lines

Spin exchange interaction usually contributes to transverse relaxation of alkali metals in a magnetometer^18^, and thus the magnetic resonance lines are broadened^13^. However, in a vapour cell with a high density of alkali metals under the condition of a low magnetic field^10^, such that the spin exchange collision rate of the pairs of alkali metals 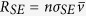 is much larger than the Larmor precession rate of the alkali metal spin *ω*_*F*=*I*±1/2_ = ±(*g*_*s*_*μ*_*B*_*B*)/(2*I* + 1)/*ħ*, the expectation value 〈*m*〉_*F*_ in the two hyper-fine states becomes locked by both rapid spin exchange and precession in the same direction; thus, spin exchange relaxation vanishes for a zero magnetic field^12^. In the equation above, *n* is the number density of an alkali metal, *σ*_*SE*_ is the spin exchange cross section between the pairs of alkali metal spins, 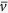 is the relative velocity between the pairs of alkali metal atoms, *g*_*s*_ is the g factor, *μ*_*B*_ is the Bohr magneton, and *I* is the nuclear magnetic moment of an alkali metal atom. A full treatment of the system requires the use of density matrix theory(see, for example, ref. 19). In the above rapid spin exchange case, the density matrix assumes a spin temperature distribution^11^ and the ground states of the alkali metal can be well described by Bloch equations^1^,^8^. The Rb and ^21^Ne spin ensembles coupled together through spin exchange interaction. In a spherical cell this coupling can be represented by an effective magnetic field that one spin species experiences from the average magnetization of the other, **B** = *λ***M**, where *λ* = 8/3*πκ*_0_^20^ and *κ*_0_ is approximately 36 for Rb and ^21^Ne pair^14^. The full Bloch equations are described as follows:


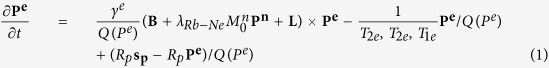



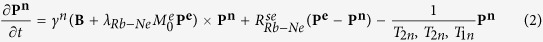


Here **P**^**e**^ and **P**^**n**^ are the Rb electron and ^21^Ne nuclear polarizations, **B** is the external magnetic field, 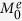 and 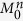 are the magnetizations of the electron and nuclear spins corresponding to full spin polarizations, **L** is the lightshift experienced by Rb atoms, **s**_**p**_ is the optical pumping vector along the propagation of the pump with magnitude equal to the degree of circular polarization, *R*_*p*_ is an effective pumping rate which is related to the K pumping rate and the density ratio of K to Rb, 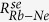 is the spin exchange rate of ^21^Ne nuclear spins. *T*_1_ and *T*_2_ are the relaxation times for appropriate for components of the polarization parallel and transverse to **B**, respectively. The subscipts *e* and *n* denote for the electron spins and nuclear spins. The precession frequency of the alkali metal is slowed by factor *Q*(*P*^*e*^)^11^, which is related to the alkali spin polarization:


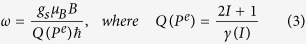


Here *ω* is the precession angular rate of the alkali spins under a magnetic field *B*, *γ*(*I*) is the dimensionless gyro-magnetic ratio. For ^85^Rb and ^87^Rb atoms, the nuclear magnetic moments are respectively *I* = 5/2 and *I* = 3/2. The dimensionless gyro-magnetic ratios are given by^11^:


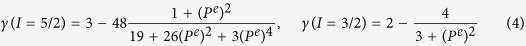


The longitudinal relaxation rate of the electron spins 1/*T*_1*e*_ is mainly come from the Rb-Rb spin destruction collision and can be written *R*_*sd*_. The transverse relaxation rate of electron spins 1/*T*_2*e*_ can be written 
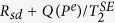
. 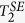 is the spin exchange relaxation between Rb and Rb collisions. In the limit of the rapid spin exchange condition, spin exchange relaxation contributes to transverse relaxation only in the second order^10^:


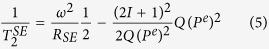


According to equation (5) and equation (3), spin exchange relaxation is correlated with the magnetic field, which is experienced by alkali metals and vanishes for a zero magnetic field. In an atomic co-magnetometer based on K-Rb-^21^Ne, we found that Rb atoms experienced a large magnetic field caused by a large Rb magnetization field^15^. The magnetization field proportional to the Rb polarization contributed to spin exchange relaxation of Rb atom spins and thus broadened the zero magnetic resonance lines.

In a K-Rb-^21^Ne co-magnetometer, ^21^Ne atoms experience a magnetic field *B*^*e*^ ≈ 100 nT through spin exchange interaction^14^ with Rb atoms and Rb atoms experience a magnetic field *B*^*n*^ ≈ 500 nT through spin exchange with ^21^Ne atoms, as the polarization of Rb is approximately 50%. As shown in Fig. 3, Rb atom spins experience a magnetic field whose value is equal to that of the Rb magnetic field experienced by ^21^Ne atoms. According to equation (5), the magnetic field experienced by Rb atoms can cause the relaxation of Rb atom spins and thus broaden the zero magnetic field resonance lines. Broadening of the magnetic resonance lines of Rb atoms in the co-magnetometer is studied in this paper. First, we measured the magnetic field of Rb atom spins at various Rb polarizations. Then we measured the spin exchange relaxation rates of Rb atom spins, and the measured data were fitted using equation(5) to study the spin exchange relaxation.

The Rb electron magnetic field is measured by the slow and fast decays of the coupled Rb and ^21^Ne ensembles(The details will be shown in the following subsection and Fig. 6(c) shows the results). Figure 4(c) shows the measured Rb magnetic field. The Rb polarization increases with the pumping power density, and thus the Rb magnetic field increases. This magnetic field is the main source field that causes spin exchange relaxation of Rb atoms.

The Rb spin exchange relaxation rates are acquired by measuring the zero field resonance lines of Rb atoms in the co-magnetometer^13^. Through the magnetic resonance lines, we can obtain the total relaxation rate composed of the pumping rate *R*_*p*_, Rb spin destruction rate *R*_*sd*_^21^, and spin exchange relaxation rate 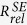 which is equal to 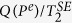
11,12. Suppose that a *B*_*y*_ step magnetic field is applied to the co-magnetometer and Rb atoms only experience *L*_*z*_ and *B*_*z*_, according to equation (1) and (2), output step modulation response signals 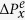 are given by:


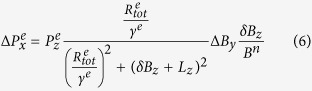


where 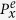 and 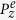 are the Rb polarizations in the x- and z-directions, *γ*^*e*^ and *γ*^*n*^ are the gyro-magnetic ratios of the electron and ^21^Ne nuclear spins, respectively, 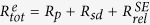 is the total relaxation rate of Rb atoms, and *δB*_*z*_ = *B*_*z*_ − *B*^*e*^ − *B*^*n*^. 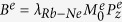 is the Rb magnetic field experienced by ^21^Ne atoms, and 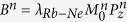 is the ^21^Ne magnetic field experienced by Rb atoms^8^,^22^,^23^. According to equation(6), the response of the co-magnetometer to *B*_*y*_ step magnetic field modulation changes with the *B*_*z*_ magnetic field(zero field resonance) and thus the total Rb relaxation rate can be measured.

Figure 4(a) shows the results of the curves fitted to equation (6) at various pumping laser power densities. The total relaxation rates of Rb atoms can be calculated using the linewidth of the zero magnetic field resonance curves in Fig. 4(a), and we summarize them in Fig. 4(b). As the pumping laser power density is smaller than approximately 0.4 V, the total Rb relaxation rate decreases rapidly. We believe that this is due to the reduction of the large Rb magnetic field *B*^*e*^. The reduction of the magnetic field experienced by Rb electron spins suppresses the spin exchange relaxation rate of Rb-Rb collisions^11^. As the pumping laser power density is higher than 1 V, the total relaxation rate deviates from a rapid increase and seems to be saturated. We think that this is due to an increase in the Rb polarization at high power densities^11^.

In order to obtain the spin exchange relaxation rate, we subtract the pumping rates from the total relaxation rates. The residual relaxation rates are the spin destruction rates *R*_*sd*_, and the spin exchange relaxation rates 
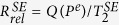
. The pumping rates are approximately proportional to the pumping power density. We obtained the pumping rates by measuring the co-magnetometer response. When *R*_*p*_ was equal to 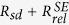, the co-magnetometer response was largest and the Rb polarization was 50%.

To acquire the maximum response of the co-magnetometer, we first showed the relationship between the output signal and the input rotation rates. In a K-Rb-^21^Ne co-magnetometer, the coupling of rotations in the x- and y-axes is significant owing to the AC Stark shift *L*_*z*_ caused by a pumping laser with a wavelength of 770 nm at the 780 nm Rb D2 line^24^,^25^. After suppressing systematic errors, such as magnetic fields in all the directions and probe laser light shift, we could obtain the relationship between the rotations and the Rb polarization in the x-direction.


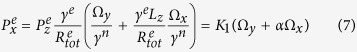


In equation (7), 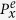 and 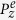 are the Rb polarizations in the x- and z-directions, respectively, *γ*^*e*^ and *γ*^*n*^ are the gyro-magnetic ratios of the electron and ^21^Ne nuclear spins, respectively, 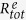 is the total relaxation rate of Rb atoms, and Ω_*y*_ and Ω_*x*_ are the rotation velocities in the y- and x-directions, respectively. *K*_1_ is the factor that converts the rotation rate signal into the x-polarization detected by the PEM system. *α* is the coupling coefficient which is related to *L*_*z*_ and Ω_*x*_.

The factor 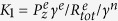 in equation (7) is measured in this experiment to optimize the response. From equation (7), we express *K*_1_ in the way:


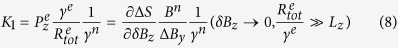


∂Δ*S*/∂*δB*_*z*_ can be calculated from the fitted Lorentz curve in Fig. 4(a). *B*^*n*^ is approximately equal to *B*_*z*_, which is directly measured in the *B*_*y*_ modulation process. Figure 5(a) shows the relationship between the pumping laser power density and the factor *K*_1_. As the pumping laser power density is approximately 1.3 V, *K*_1_ in the co-magnetometer is the best. The Rb polarization is approximately 50% at 1.3 V. This indicates that the co-magnetometer response is largest when the Rb polarization is approximately 50%. Although the response in Fig. 5(a) is considerably large when the pump power density is smaller than 0.25 V, the polarization of Rb is considerably small and leads to a small ^21^Ne polarization. A smaller ^21^Ne polarization leads to an inferior co-magnetometer performance of the suppression of external magnetic field noise. According to Fig. 4(b), the total relaxation rate of Rb atoms is approximately 3900 s^−1^ at 1.3 V. We can conclude that the pumping rate of Rb atoms is approximately 1950 s^−1^. The pumping rate is 0 when the pumping power density is 0. We know that the relationship between the pumping power density and the pumping rate is *R*_*p*_ = 1500 s^−1^/V × *Power*. *Power* is the input power density of the laser. We subtract the pumping rate from the total relaxation rate of Rb atoms in Fig. 4(b) and combine the measured Rb magnetic field at various pumping power densities. We can obtain the relationship between the Rb magnetic field and the spin relaxation rate 
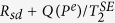
. Figure 4(d) shows the measured results. We fit the measured data to 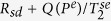. In this experiment, natural abundance Rb is used and 72% of Rb atoms are ^85^Rb with nuclear spin *I* = 5/2. We suppose that Rb atoms are mainly ^85^Rb atoms for approximation. The fitted results show that the spin destruction rate *R*_*sd*_ is 482 s^−1^. This result is consistent with the calculated spin destruction rate of Rb-Rb collisions and Rb-^21^Ne collisions (the total spin destruction rate is 477 s^−1^) in the co-magnetometer if the temperature of the vapour cell is 458 K. In equation (5), we suppose that the polarization of Rb is *kB*^*e*^. We can also obtain coefficient *k* ≈ 0.008. When the polarization of Rb is 1, the magnetic field of Rb electron spins can be calculated from *k* to be 125 nT. The spin exchange rate *R*^*SE*^ is fitted to be approximately 1.6 × 10^5^ s^−1^. In the co-magnetometer, the spin exchange rate between Rb atoms 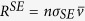 is calculated to be 4.5 × 10^5^ s^−1^. We believe that the difference between the calculated and fitted results is from the approximation that only ^85^Rb is used. A compound will broaden the zero magnetic field resonance lines of the co-magnetometer. We also simulated the spin relaxation rate of Rb atoms in the co-magnetometer when pure ^87^Rb atoms with nuclear spin *I* = 3/2 were used. The theoretical prediction in Fig. 4(d) shows that the total spin relaxation rate of ^87^Rb atoms is smaller than the relaxation rate of ^85^Rb atoms when the Rb magnetic field is about 100 nT.

The pumping power density can be optimized to improve the co-magnetometer sensitivity by studying the spin exchange relaxation rate of Rb atoms. The co-magnetometer response to the angular velocity should be largest if the pumping rate is equal to the relaxation rate of Rb atoms. The polarization of Rb should be 50% at this state. Figure 5(a) shows the relationship between the co-magnetometer response and the pumping power density. As the pumping power density is approximately 1.3 V, the co-magnetometer response is largest and the polarization of Rb is approximately 50%. We chose a pumping power density of 1.3 V for the co-magnetometer.

### Sensitivity optimization

The co-magnetometer sensitivity was optimized by maximizing the co-magnetometer response to the angular velocity and suppressing noises of the co-magnetometer. For maximizing the response, we mainly optimized the pumping power density (the optimization is shown in the Rb magnetic resonance line broadening section). For noise suppression, we mainly reduced the magnetic field noise by optimizing the self-compensating ability and utilizing a low magnetic field noise shielding material for the innermost layer. Laser power fluctuation noise suppression was also studied.

#### Magnetic field noise suppression optimization

Rb atoms and ^21^Ne atoms occupied the same volume and coupled together to suppress the co-magnetometer sensitivity to magnetic field noise and ensure it remains sensitive only to the rotation rate, etc. The suppression factor used to characterize the co-magnetometer ability to suppress its sensitivity to magnetic field noise was measured in this study. We optimized the temperature of the vapour cell to improve the suppression factors. In an atomic co-magnetometer, the axis most sensitive to the magnetic field is in the x-direction^1^,^15^. When sinusoidal magnetic field noise *B*_*x*_sin(2*πft*) in the x-direction with frequency *f* is supposed to be applied to the co-magnetometer, the output signal is^15^:


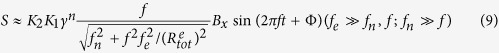


where *f*_*n*_ = *γ*^*n*^*B*^*n*^ and *f*_*e*_ = *γ*^*e*^*B*^*e*^. Φ is the phase shift between the input magnetic field and the output signal. *K*_2_ is the factor that converts the Rb polarization in x-direction into the output voltage signal by the PEM detection system. Therefore, the combination of *K*_1_*K*_2_ is the scale factor of the co-magnetometer. The suppression factor is defined to be:


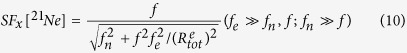


According to equations (10) and (7), we know that the co-magnetometer sensitivity to *B*_*x*_ is suppressed by the suppression factors. We applied a sinusoidal magnetic field in the x-direction and measured the output signal amplitude to acquire the suppression factors. Figure 6(a) shows the *B*_*x*_ suppression factors at different temperatures. To fit the measured data to equation (10), *f*_*n*_ = *γ*^*n*^*B*^*n*^ must be measured. *B*^*n*^ is the magnetic field generated by ^21^Ne atoms. We developed a simple method to measure *B*^*n*^. The result is illustrated in Fig. 6(c). As the compensation magnetic field in the z-direction *B*_*z*_ is tuned to −*B*^*n*^ − *B*^*e*^, the co-magnetometer can automatically compensate *B*_*y*_^8^. Slow decay in Fig. 6(c) shows the transient response of the co-magnetometer to the step magnetic field *B*_*y*_ when *B*_*z*_ is equal to −*B*^*n*^ − *B*^*e*^. When *B*_*z*_ is approximately equal to −*B*^*n*^ the coupled Rb and ^21^Ne spin ensembles decay most rapidly. Fast decay in Fig. 6(c) shows the transient response of the co-magnetometer to the step magnetic field *B*_*y*_ if *B*_*z*_ is equal to −*B*^*n*^. Figure 6(b) shows the relationship between the Rb densities and the Rb polarization at the same pumping power under various temperatures. The Rb polarization was measured to ensure that the polarization of Rb was approximately 50% as the temperature of the vapour cell was increased. The measured results in Fig. 6(b) show that the polarization of Rb changes slightly and is approximately 50% as the temperature of the vapour cell is increased. To measure the Rb polarization, the fitted results in Fig. 6(a) are used to obtain the Rb relaxation rate 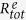 in equation (10). The pumping rate combined with the total relaxation rate can be used to obtain the Rb polarization. As the temperature increases, the polarization of Rb spins decreases. This is explained by an increase in the relaxation rate of Rb spins with the temperature.

From Fig. 6(a), we can conclude that the suppression factor can be further enhanced by increasing the co-magnetometer temperature. In this experiment, the temperature of a heater was difficult to be increased owing to a technical reason. The temperature of the vapour cell was approximately 473 K, and the heater could suffer from the maximum temperature of approximately 493 K. A new type of heater that can sustain a high temperature needs to be developed to further enhance the suppression factors. In addition, the pumping laser power works at its maximum power output. The polarization of Rb decreased to approximately 0.4. As we increased the temperature of the vapour cell, the pumping power was also needed to be increased to reach the 50% polarization state.

#### Laser related noise suppression

The scale factor *K*_1_*K*_2_ is directly related to the probe laser power and frequency. The input of the rotation rate of the earth gives signal *S* = *K*_2_*K*_1_(Ω_*y*_ + *α*Ω_*x*_). Fluctuations of the probe beam power and frequency produce noise in the output signal through the coupling term of the rotation rate. Thus, we reduced the input of the rotation rate projected by the earth to lower the noise. We designed a rotating co-magnetometer to suppress this kind of noises. In this study, the rotating co-magnetometer sensitive axis was fixed to the east–north direction to suppress the input of the earth’s rotation. The co-magnetometer is supposed to be at a north latitude of Ψ. *θ* is the angle between the sensitive y-axis of the co-magnetometer and the east direction. The relation between *θ* and the co-magnetometer output signal is:


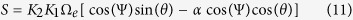


In equation (11), Ω_*e*_ is the rotation of the earth. We could change *θ* to let the output signal *S* to be 0. To do this, we measured the rotation of the earth using the co-magnetometer; the result is shown in Fig. 7(a). We know that the output signal is 0 if the axis is set to 13.0° in the east–north direction. This is due to coupling of the two axis input rotation rates through *L*_*z*_ in equation (11). The fitted curve in Fig. 7(a) shows that the coupling factor *α* is 0.23. Details of the earth’s rotation measurement are presented in the methods’ section. The earth’s rotation measurement can also be utilized to calibrate the co-magnetometer.

Pumping laser power density fluctuation can also produce noise in the co-magnetometer. It can lead to fluctuations of the Rb pumping rate for *R*_*p*_ = Φ*σ*(*ν*), where Φ is the photon flux, which is directly related to the pumping power density and *σ*(*ν*) is the collision cross section of a photon and a Rb atom. Misalignment of the pump and probe beams by angle *β* away from 90° gives signal 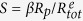. If the angle *β* is 0, pumping power fluctuation has no effect on the output signal. The reduction of the angle *β* suppresses the co-magnetometer sensitivity to pumping power fluctuation. In this study, we adjusted the pump beam using two mirrors to vary the angle *β*. We measured the output signal of the co-magnetometer as the pumping laser was turned on and off. We adjusted the angle *β* until the two output signals were coincident. The residual magnetic fields should be compensated when the angle *β* is adjusted.

### Rotation rate sensitivity measurement

The sensitivity of the co-magnetometer was measured after co-magnetometer calibration. The output signal was acquired by means of National Instrument (NI) 24 bit data acquisition systems. The power spectral density of the output signal was analysed to measure the noise level of the co-magnetometer. The power spectral density was averaged in each 0.1 Hz bin. Scale factor *K*_2_*K*_1_ was used to convert the output voltage signal into the rotation rate signal. The co-magnetometer sensitivity is illustrated in Fig. 7(b). The rotation velocity noise is 2.1 × 10^−8^ rad s^−1^ Hz^−1/2^, which is equal to a magnetic field noise sensitivity of 1.0 fT Hz^−1/2^. The two peaks between 1 Hz and 5 Hz in Fig. 7(b) are the co-magnetometer responses to vibrations of the isolation platform. The decrease of the noise below 10 Hz is caused by setting the cut off frequency of the filter to 10 Hz in the PEM measurement system.

## Discussion

In a co-magnetometer based on SERF, alkali metal atoms are in the SERF regime to sensitively detect the direction of noble gas nuclear spins. Traditionally, spin exchange relaxation is neglected in a co-magnetometer. This study shows that in a K-Rb-^21^Ne co-magnetometer, the magnetic resonance lines are broadened through spin exchange relaxation. The detail study shows that spin exchange relaxation is caused by spin exchange interaction between Rb and ^21^Ne spins which produces a large Rb magnetic field that in turn increases the Larmor precession frequency of Rb atoms which increases spin exchange relaxation. We modelled this process in the K-Rb-^21^Ne co-magnetometer. Figure 4(d) shows that the spin exchange relaxation rate of Rb-Rb collisions^11^ increases rapidly with the Rb magnetic field. Natural abundance Rb is used in the co-magnetometer, and Rb atoms are mostly ^85^Rb atoms with nuclear spin *I* = 5/2. It is actually a K-^87^Rb-^85^Rb-^21^Ne co-magnetometer. We found that if pure ^87^Rb atoms with nuclear spins *I* = 3/2 were used, the spin exchange relaxation rate was suppressed and thus the co-magnetometer sensitivity could be improved. We implemented a model based on the hypothesis that Rb atoms used in this study were pure ^85^Rb atoms (actually, 72% of Rb atoms were ^85^Rb atoms). Models should be developed to simulate the spin exchange relaxation rate of natural abundance Rb atoms which contain both ^87^Rb and ^85^Rb. Alkali vapour cell fabrication of a K-^87^Rb-^21^Ne cell is under design. In order to control the mole fraction ratio of K to Rb and save the expensive isotope enriched ^87^Rb metal, a chemical reaction method is under design^26^.

The sensitivity of a co-magnetometer for rotation velocity sensing determines the angle random walk of a rate sensor. For anomalous field or electric dipole moment detection, the co-magnetometer sensitivity needs to be strengthened to detect such weak signals. The co-magnetometer described in this paper is optimized by enlarging the response and suppressing various noises. In order to maximize the co-magnetometer response, we optimized the pumping laser power density. The co-magnetometer response is largest when the relaxation rate of Rb is equal to the pumping rate.

The magnetic field noise can be further suppressed by optimizing the self-compensating suppression factors of the co-magnetometer. As can be seen in Fig. 6(a), we can further increase the cell temperature to improve the suppression factors. Simultaneously, the pumping laser power should be increased to reach the best power density point. In this experiment, the largest output power of the TA was 1 W, which is insufficient for higher-temperature vapour cells. In the next generation co-magnetometer design, we can use a smaller cell or develop a higher-power tapered amplifier to optimize the suppression factor. On the other hand, not only the co-magnetometer itself can suppress the magnetic field noise, but also we can use a ferrite with a larger diameter to reduce the magnetic noise produced by the shielding material itself^17^ (currently, the ferrite used in this experiment has a diameter of approximately 100 mm).

The ultimate precision limit of the co-magnetometer is determined by quantum principles. Spin projection noise, photon shot noise, and light-shift noise are typical quantum noises in a co-magnetometer. Let us take spin projection noise for example in the co-magnetometer studied in this paper. It originates from the uncertainty of the x-component of angular moment *F*_*x*_. The angular moment *F*_*x*_ does not commutate with *F*_*y*_^13^. For independent probes, the ultimate precision limit is proportional to 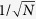 if the probes are not entangled. For noiseless processes, by entangling the probes, the ultimate precision limit which scales with 1/*N*, the so-called Heisenberg limit, can be achieved. For noisy quantum enhanced metrology, the general framework for estimating the ultimate precision limit was studied by Escher, Filho, and Davidovich^27^. Brask, Chaves, and Kołodyński argued that the transversal-noise model, which can be used in noisy quantum enhanced metrology, can be applied to atomic magnetometry. It is accessible with current experimental techniques^28^. Moreover, in an SERF co-magnetometer or the magnetometer studied in this paper, Liu, Cheng, and Qi *et al*. demonstrated how spin-destruction collisions degrade the precision from the Heisenberg limit to the standard limit even with quantum resources being employed for static magnetic fields^29^. Even though the co-magnetometer sensitivity is limited by technical noises, the methods related to the sensitivity limit are worth discussing. The sensitivity can be further improved by implementing these methods after classical technical noise reduction.

## Methods

### Calibrating the co-magnetometer

**Figure 2:**
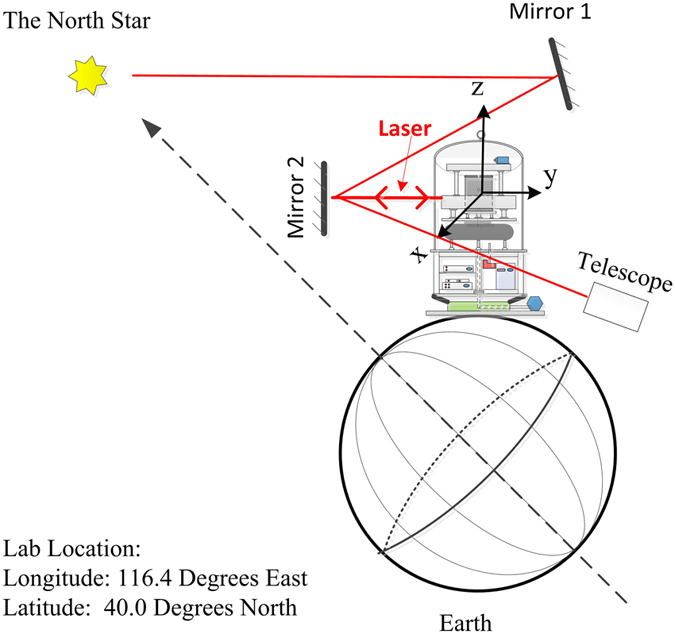
Schematic of the calibrating northern direction using the North Star. A high-precision encoder is on the rotary platform, and the platform heading can be defined using the encoder. There is a mirror (Mirror 2) in our laboratory whose vertical direction is calibrated using the North Star. The vertical direction of the mirror plane is the north direction. A laser beam is parallel to the co-magnetometer x-axis to be incident on Mirror 2, as the reflected beam is coincident with the input beam. The heading degree on the encoder is the north direction.

**Figure 3:**
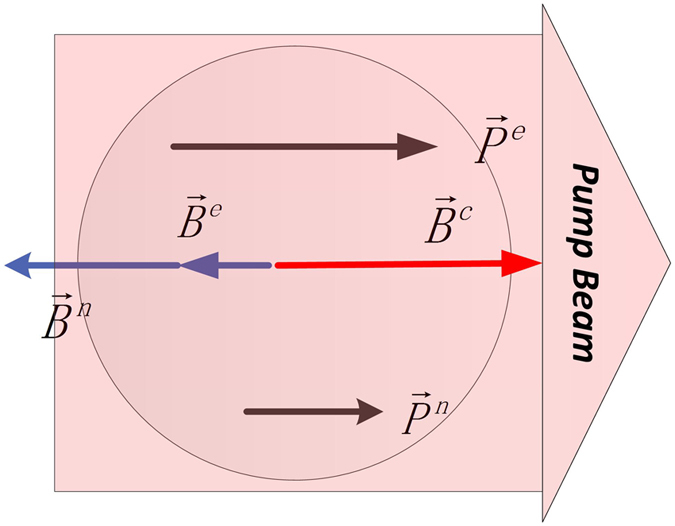
Schematic of the polarization and the magnetic field of Rb and ^21^Ne spins. Superscripts *e* and *n* denote the electron spin of Rb and the ^21^Ne nuclear spin, respectively. The pump beam polarizes the Rb electron spin to *P*^*e*^, and the nuclear spins are polarized by the electron spins to be *P*^*n*^. The nuclear spin experiences the magnetic field *B*^*e*^ produced by *P*^*e*^, and the electron spins experience the magnetic field *B*^*n*^ produced by the nuclear spins. A magnetic field *B*^*c*^, which is equal to *B*^*e*^ + *B*^*n*^, is applied to reach the magnetic field self-compensation state. Thus, the electron spins experience the magnetic field *B*^*e*^ which increases the spin exchange relaxation rate of the Rb atom spins.

**Figure 4:**
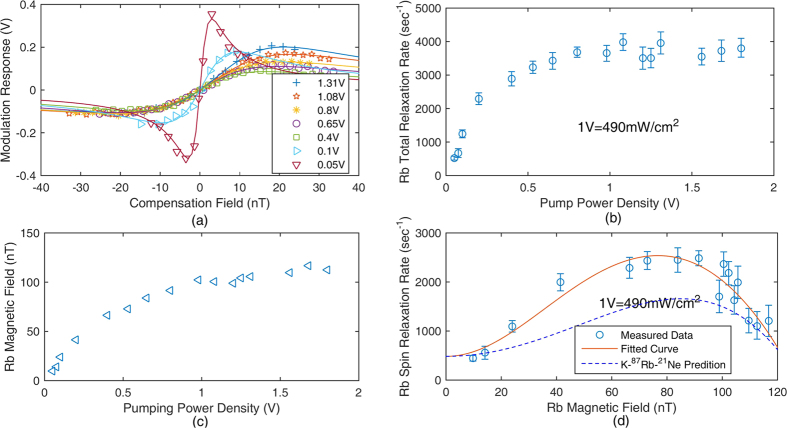
(**a**) Zero magnetic field resonance lines of the co-magnetometer which is used to obtain the total relaxation rates of Rb atom spins for various power densities. The *B*_*y*_ modulation method is used to evaluate the total relaxation rate of Rb electron spins. According to equation (6), the response signal changes with the holding magnetic field *B*_*z*_. The relationship between *δB*_*z*_ and the modulation response signal is fitted by equation (6) at various pumping laser power densities (1 V = 490 mW/cm^2^). The fitted curve is a Lorentz-shaped curve, and the half width at half maximum linewidth of the curve is 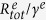, which is the total relaxation rate of Rb spins. (**b**) The Rb total relaxation rate measurement at various pumping power densities. As the pumping power is small or large, the spin exchange relaxation rate is suppressed. (**c**) The measured Rb magnetic field at various pumping power densities. The Rb magnetic field can broaden the zero magnetic field lines by increasing the spin exchange relaxation rate of Rb atoms. The Rb magnetic field increases with the pumping power. (**d**) The relationship between the Rb magnetic field and the spin relaxation rate 
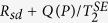
, where *R*_*sd*_ is the spin destruction rate. The measured data are fitted to the theoretical prediction. The spin relaxation rate is strongly related to the Rb magnetic field, which can broaden the zero magnetic resonance lines of the co-magnetometer. The theoretically predicted spin relaxation in the pure ^87^Rb co-magnetometer is also illustrated. We know that if we change natural abundance Rb atoms into pure ^87^Rb atoms in the vapour cell, the spin relaxation rate can be reduced as the Rb magnetic field is about 100 nT, which corresponds to a polarization of Rb atoms of approximately 50%.

**Figure 5:**
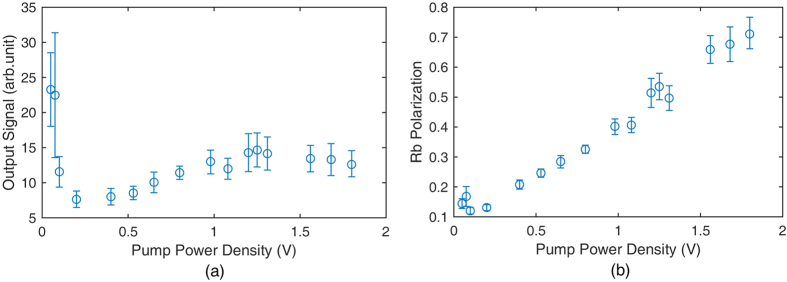
(**a**) Relationship between the co-magnetometer response and the pumping power density. The co-magnetometer response is modelled to be *K*_1_. The optimized power density is 1.3 V, and the co-magnetometer response is largest. Although the responses at very small power densities are larger than that at 1.3 V, these power densities are not appropriate, as the ^21^Ne polarization is too small to suppress the magnetic field. (**b**) The measured Rb polarization at various power densities. The Rb polarization is calculated using the pumping rate and the total relaxation rate. Both rates are measured in Fig. 4(b). As the pumping laser power density is approximately 1.3 V, the Rb polarization is approximately 50%. Simultaneously, the co-magnetometer response is largest.

**Figure 6:**
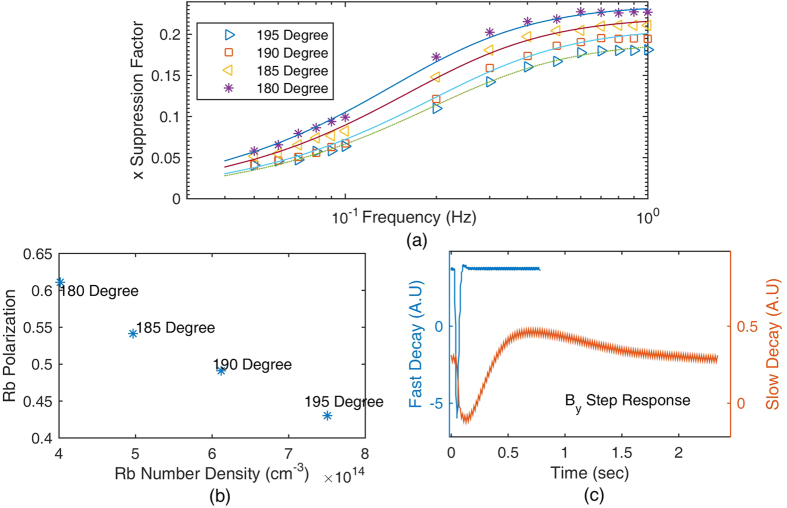
(**a**) *B*_*x*_ suppression factors at various temperatures. The suppression factors describe the co-magnetometer ability to suppress magnetic field noise. A smaller suppression factor indicates that the magnetic field is better suppressed by the co-magnetometer. The suppression factors can be fitted to the theoretical results. The suppression factors are better when the temperature of the vapour cell is higher. (**b**) The Rb polarization changes with the cell temperature while the pumping power density is 1.3 V. When the temperature is optimized, the polarization of Rb atoms is also measured to confirm that the polarization of Rb atoms is approximately 50%. As the number density of Rb atoms changes from 4.0 × 10^14^ cm^−3^ to 7.5 × 10^14^ cm^−3^, the polarization of Rb changes from approximately 0.6 to 0.4. This result shows that the polarization of Rb is approximately 50%. The co-magnetometer responses to rotations are almost largest. (**c**) The decays of the coupled spin ensembles to the step magnetic field *B*_*y*_ for different compensating fields *B*_*z*_. These results are used to get *B*^*n*^ and *B*^*e*^ to fit the suppression factor curve in Fig. 6(a). We tune *B*_*z*_. The spins decay most rapidly if *B*_*z*_ is equal to −*B*^*n*^. At the self-compensation point, *B*_*z*_ is equal to −*B*^*n*^ − *B*^*e*^. The slow decay in this figure shows the co-magnetometer response to the step magnetic field *B*_*y*_ at the self-compensation point.

In equation (7), the co-magnetometer is sensitive to the rotation rates. The projected rotation rates of the earth in the x- and y-directions change as the co-magnetometer sensitive axis changes in the horizontal plane. We used the North Star as navigation reference to find the north direction. In Fig. 2, the North Star light is reflected by Mirror 1 and Mirror 2 to a telescope. Mirror 2 is screwed, and the vertical direction to the plane of Mirror 2 is fixed to be parallel to the north direction. A laser is mounted on the co-magnetometer platform, and the laser beam direction can be changed to be parallel to the co-magnetometer y-direction. We changed the co-magnetometer y-direction to let the laser light reflected from Mirror 2 to coincide with the incident laser light. Thus, the y-direction was in the north direction.

**Figure 7:**
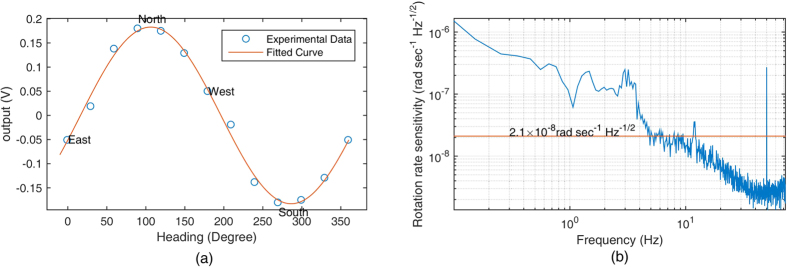
(**a**) Co-magnetometer calibration result by the rotation of the earth. The measurement was started from the east direction. The sensitive axis in the y-direction is heading to various directions at every 30°. We measured the output signal at one heading position and then measured it at the opposite direction. This method could efficiently reduce the co-magnetometer drift. (**b**) The measured rotation rate sensitivity of the co-magnetometer. The co-magnetometer output signal was obtained, and the power spectral density was calculated. The scale factor calibrated by the rotation of the earth was used to convert the voltage signal into the rotation rate signal. The peaks between 1 Hz and 5 Hz are caused by the vibration isolation platform. The platform is a mechanical vibration system. The whole payload on the platform is about 250 kg.

After we found the north direction, the co-magnetometer was calibrated by the rotation of the earth. According to equation (7), the co-magnetometer is sensitive to both y- and x-rotation rates. We started the calibration by fixing the co-magnetometer sensitive axis to the east direction. The reading of the encoder on the direct drive rotation platform was set to 0° as the y-axis was in the east direction. After measuring the output signal *V*_0_ = *K*_2_*K*_1_Ω_0_ + *V*_*background*_ at a 0° position, we rotated the y-axis to a 180° position. Then the output signal *V*_180_ = *K*_2_*K*_1_Ω_180_ + *V*_*background*_ was measured. *K*_2_ is the factor that converts the polarization in the x-axis into the output voltage signal by the PEM measurement system. Thus, *K*_2_*K*_1_ is the scale factor which converts the rotation rate signal into the output voltage signal. *V*_*background*_ is the background output signal as there are no input rotation rates. From the description above we know that Ω_0_ is equal to −Ω_180_. The rotation rate Ω_0_ is calculated to be (*V*_0_ − *V*_180_)/2. This method can significantly reduce the drift of the co-magnetometer as we measured the projection of the earth’s rotation rate. Figure 7(a) shows the calibration results when the sensitive axis heading in different directions. The residual magnetic fields in the three directions were compensated in each heading direction to suppress the signal responses to magnetic fields. The measured rotation rates were fitted to equation (11). The co-magnetometer calibration result by the rotation of the earth is 1576 ± 92 V/(rad/s).

The co-magnetometer can also be calibrated by the modulation magnetic field *B*_*x*_. In equation (9), sinusoidal magnetic field *B*_*x*_sin(2*πft*) is applied to the co-magnetometer, and the output signal amplitude is measured. The measured signal was fitted to equation (9), and then the scale factor *K*_1_*K*_2_ could be acquired. This process could also calibrate the co-magnetometer. We applied a magnetic field of approximately 0.2 nT_*pp*_ in the x-direction. The measured scale factor is 1610 ± 138 V/(rad/s). By comparing the calibration result through the earth’s rotation to the *B*_*x*_ modulation method result, we obtained similar calibration results. We used the average calibration result of the two methods as the final calibration result, and the final calibration result is 1590 V/(rad/s).

### Sensitivity measurement details

**Figure 8:**
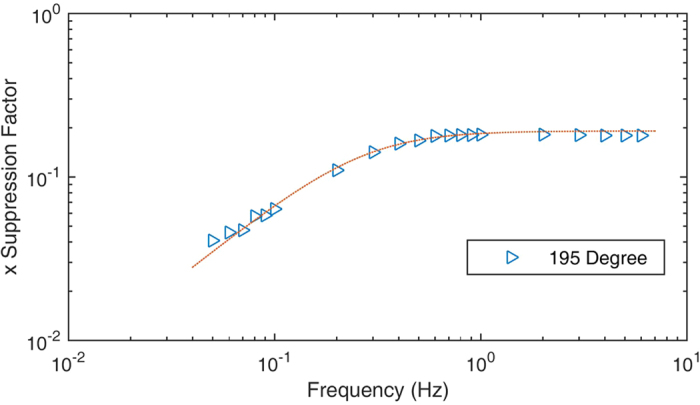
Relationship between the input magnetic field *B*_*x*_ frequencies and the suppression factors. This figure also shows the response of the co-magnetometer to the magnetic field, which can be utilized to obtain the co-magnetometer sensitivity to the magnetic field at different frequencies.

The co-magnetometer sensitivity was obtained by measuring the output signal noise. As there was no rotation velocity input, technical noises, such as laser position noise, laser power fluctuation noise, and magnetic field noise, dominated the output signal. To measure small rotations, the noise level must be lowered. The power spectral density of the output signal was calculated to acquire the root mean square voltage at different frequencies. As the signal is the voltage signal, the output voltage signal should be converted into the magnetic field or rotation velocity units. Similar to an SERF magnetometer^12^, the co-magnetometer bandwidth is several hertz. In this study, the compensation magnetic field is approximately 600 nT, which is approximately equal to the magnetic field of ^21^Ne spins. The precession frequency of ^21^Ne spins is approximately 2 Hz. At the compensation magnetic field point, the compensation magnetic field of ^3^He in the K-^3^He co-magnetic field is approximately 20 Hz^16^. These precession frequencies indicate that the co-magnetometers have a limited bandwidth. A response curve should be measured to obtain the scale factors of the co-magnetometer at different frequencies^12^. In an atomic co-magnetometer based on SERF, the co-magnetometer is more sensitive to the magnetic field in the x-direction than to that in other directions. Therefore, we only measured the co-magnetometer response to the magnetic field in the x-direction. According to equation (9), if a sinusoidal magnetic field with frequency *f* is applied in the x-direction, the co-magnetometer response to this magnetic field can be solved and measured. We measured constant scale factor *K*_2_*K*_1_ and the suppression factors which are defined in the equation. Figure 8 shows the suppression factors that are proportional to the co-magnetometer response to the magnetic field at different frequencies. The measured suppression factors were fitted to equation (9). As the frequencies were slower than 0.2 Hz, the suppression factors depended on the frequency linearly. As the frequencies were larger than 1 Hz, the suppression factors went into a relatively flat area. This was also verified by the studies on dynamics of Rb and ^21^Ne^30^ spin ensembles. If magnetic field noise *B*_*rms*_ with frequency *f* was applied to the co-magnetometer, the noise would be suppressed by the suppression factors at this frequency. The total magnetic field noise is *SF*_*x*_ *B*_*rms*_(2*πf*). According to equation (9), the total magnetic field noise is calculated to be *S*_*rms*_/(*K*_2_*K*_1_*γ*^*n*^). The output voltage signal was recorded using a 24 bit NI data acquire system, and the power spectral density of the output signal was acquired to measure the noise level. Figure 7(b) shows the results. The noise level could be changed to different units by the measured scale factor *K*_1_*K*_2_ and *γ*^*n*^. The noise level in Fig. 7(b) was the averaged noise level from the raw data to obtain a relatively smooth level. In each 0.1 Hz bin, the noises were averaged. The measured magnetic field noise level is 1.0 *fT*/*Hz*^1/2^@5 Hz. The rotation sensitivity of the co-magnetometer was calculated to be 2.1 × 10^−8^ rad s^−1^ Hz^−1/2^@5 Hz under the magnetic field noise level.

## Additional Information

**How to cite this article**: Chen, Y. *et al*. Spin exchange broadening of magnetic resonance lines in a high-sensitivity rotating K-Rb-^21^Ne co-magnetometer. *Sci. Rep*. **6**, 36547; doi: 10.1038/srep36547 (2016).

**Publisher’s note:** Springer Nature remains neutral with regard to jurisdictional claims in published maps and institutional affiliations.

## Figures and Tables

**Figure 1:**
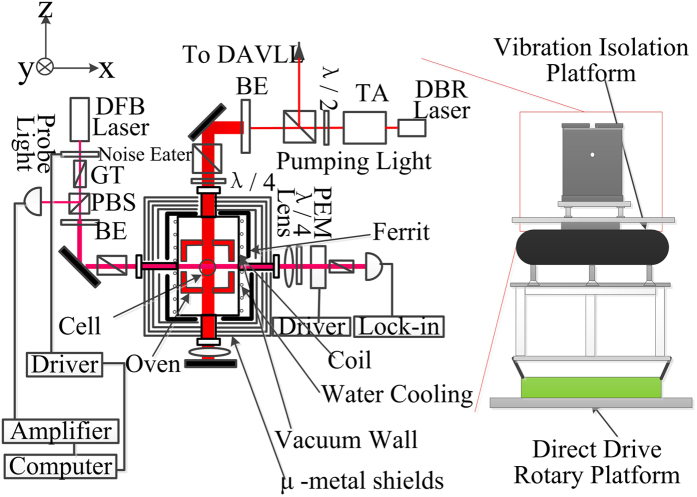
Schematic of the experimental setup. The setup is similar to the one developed by Brown, Smullin, Kornack, and Romalis^16^. The vapour cell is at the centre of the co-magnetometer. A vertical pump beam and a horizontal probe beam are used to polarize atoms and probe atom spins. The vapour cell is heated, and magnetic field shields are used to shield atom spins from magnetic fields. The core of the co-magnetometer is configured on a vibration isolation platform. A bell jar, where air fluctuation is reduced, is used to enclose the laser beam in vacuum. The apparatus is constructed on a rotary platform, which is utilized to rotate the sensitive axis of the co-magnetometer around the z-axis. To improve the sensitivity, the sensitive axis is fixed to the east direction to reduce the input rotation of the earth to approximately 0 as the co-magnetometer is constructed in our laboratory at a certain latitude and longitude in Beijing. Moreover, the co-magnetometer can be calibrated by the rotation of the earth. BE: beam expander, PBS: polarization beam splitter, GT: Glan-Thompson polarizer, PD: photo detector, PEM: photo elastic modulator, TA: tapered amplifier, DAVLL: dichroic atomic vapour laser lock, DBR: distributed Bragg reflector, and DFB: distributed feedback.
